# Cereus Bonplandii

**Published:** 1892-12

**Authors:** J. H. Fitch

**Affiliations:** New Scotland, N. Y.


### CEREUS BONPLANDII.

J. H. Fitch, M. D., New Scotland, N. Y.

*Mind and Disposition.*—An agreeable, tranquil state and frame of mind and body (first day, evening).

Mind perfectly composed.

Feel better when engaged at something or occupied.

Desire to be at useful work, desire to be busy (second day).

Desire to be employed.

Praying or disposition to be at prayer.

Ill at ease.

Restless (third day).

Doesn't know what to do with one's self.

Feels a strong desire to give away something very necessary for him to keep or have.

Feeling irritable (on rising).

Cannot keep himself employed at anything.

Very much disturbed in mind.

Passes the time in useless occupation (fourth day).

Very irritable; acts impulsively.

Spends the whole forenoon uselessly.

Difficulty in becoming devotional (at church).

Finds it easy to become devotional.

Feels well late in the evening (seventh day).

Thinks he is under a powerful influence.

*Sensorium.*—Vertigo followed by nausea.

Swimming of the head (sixth day).

*Head.*—Decidedly painful drawing sensation in the occiput, soon subsiding (first day).

Painful stunning feeling in the right frontal bone.

Pressive pain from without inward in the occiput high up on walking.

Slight painful pressure in the right occiput from behind forward (second day).

Disagreeable feeling in occiput, running down over the neck, followed by a slight qualmishness.

Slight heavy feeling in the top of the forehead.

Headache occipital, continued for a quarter of an hour.

Sensation, as if something hard like a board were bound against the back of the head, felt more especially on left side.

Head feels drawn to the left backward.

Pain in occiput running through lobes of the cerebrum.

Pain running from left ear through the head to left ear and right parietal bone.

Pain commencing in the medulla oblongata and running upward and expanding to the surface of the brain, worse on stooping or bending forward.

Pain along right external angular process of frontal bone.

Pain through or across the brain from left to right.

Feeling as of being pressed at left occiput and immediately thereafter a counter pain in left frontal bone, the latter continuing a minute or two.

Pain from left occiput verging around left parietal bone.

Pain through occiput.

Pain in right forehead (third day).

Pain in anterior portion of brain and extending in a backward direction.

Tenderness at the point of exit of the left supra-orbital nerve.

Pain in occiput (high up).

Occipital pain (fifth day).

Bad feeling, head (third day).

*Eyes.*—Pain over right eye, passing down over globe (first day).

Nauseated feeling commencing in throat, passing to stomach simultaneous with a congested feeling in both eyes.

Pain in orbits, running from before backward.

Pain in left eyelids when stooping low (second day).

On closing the eyes perception of a cluster of round-shaped, symmetrical orange-colored spots.

Swimming eyes.

Capillary congestion of the conjunctiva.

Severe photophobia, producing a sticking pain through eyes.

Sore feeling through eyes as if exposed to strong sunlight.

Pain through globe of right eye.

Pain in the globe of left eye.

*Nose.*—Greenish (pale) mucus discharged from nostril.

Accumulation of mucus in nose as in nasal catarrh.

Stinging in nose, more especially right side.

Stinging in right nostril.

Sneezing.

Hardened mucus in left nostril.

*Face.*—Pain along right malar bone running to temple.

Looks haggard.

Yellowish face or countenance.

*Mouth, etc.*—Saliva in mouth when swallowed of no unpleasant taste (first day).

Feeling of coldness in the mouth (second day).

Feeling as of having eaten something tasting alkaline.

Water in the mouth.

Metallic taste in the mouth.

Watery saliva in the mouth (not disagreeable).

Slight metallic taste, feels as if having eaten something of a metallic taste.

Taste of green vegetables.

Watery taste.

Sensation as of a thread of mucus on the tongue.

Insipid, watery taste (third day).

Fetid breath (noticed by myself) (fourth day).

Fetid breath noticed by others (fifth day).

Tongue looks frothy (sixth day).

Tongue of a purplish red hue.

Tongue feels rough.

*Throat.*—Mucus adherent to the hard palate easily removed (first day).

Mucus in pharynx easily detached (second day).

Mucus in larynx easily detached.

Scraping of mucus, which seems to adhere to left side of pharynx.

Persistent accumulation of mucus in the pharynx, continually and recurring in considerable quantities and of a pale-green color.

Mucus easily expectorated or cleared from the throat.

Clearing of hard palate of mucus.

*Stomach, Appetite, etc.*—Dry eructations (second day).

Thirstlessness.

Appetite diminished; ate very light breakfast (third day).

Relish for sweet things.

*Abdomen, Stool, etc.*—Slight rumbling in bowels, left side (first day).

Nearly or quite inefficient effort to evacuate bowels.

Fetid flatus passed from bowels.

Slight pain in epigastrium, coming and going at intervals of a few minutes.

Slightly painful sensation in epigastrium (second day).

Passed stool not easy, not sufficient at 6 A. M. (third day).

Natural stool at 6 A. M. (sixth day).

*Urine and Urinary Organs.*—Inclination to pass urine (first day).

Urine of a slightly brownish tinge (second day).

Urine smells strongly after a few minutes.

Yellowish urine.

Urine less than half usual quantity.

Urine normal.

Urine clear, small in quantity.

Urination frequent (at 4 P. M) (second day).

Amelioration after urination.

Passed a small quantity saturated yellowish urine.

*Sexual.*—Slight increase of sexual desire.

Anæsthesia and dwindling of the sexual organs.

*Kidneys.*—Slight pain of a sticking character in right kidney (second day).

Pain in left kidney, long continued, as from the presence of a renal calculus.

Pain in left abdomen sharp and cutting, as from a calculus impacted in the ureter.

Slight pain in right kidney repeated after an interval (third day).

Sticking pain in right ureter.

More severe sticking pain in right kidney.

Soreness on external pressure over right kidney.

Pain on stooping, bending over in right kidney.

Pain in left kidney (fifth day).

*Chest, Heart, etc.*—Deep inspiration as if tired, although experiencing no fatigue whatever (second day).

Feels as if pained or oppressed at chest.

Slightly painful sensation at left chest, region of the heart.

Deep inspiration.

At intervals deep inspiration, as if the chest were laboring under an oppression hardly definable.

Slight feeling of oppression, or a weakness in the chest with the deep inspiration.

Tendency to expand the chest automatically and rhythmically, recurring very frequently.

The chest expands itself to its utmost capacity seemingly, and in an instant collapses, the same process to be repeated.

Respiration measured, no interval between inspiration and expiration.

Sensation of uneasiness extending to lumbar region on deep inspiration (described above).

Slightly pricking sensation of pain in the heart.

Sighing respiration (very frequent) (fourth day).

Tenderness of the anterior lower left intercostal muscles below the heart (third day).

Pain in chest and through heart, with pain running toward spleen, the latter momentarily, the former (heart pain) continuing.

Pain in left great pectoral muscle, worse toward the tendon.

Sighing respiration, noticed many times (fifth day).

Coughing on throwing off outer garments.

Somewhat persistent pains in the cartilages of the left lower ribs.

Long, deep, uneasy respiration, felt more acutely (sixth day).

The chest acts automatically, not according to will or whim.

Chest feels empty.

Pain at heart.

Pulse dicrotic, and several intermissions noticed within a minute (after rising 6 A. M).

Deep inspiration and expiration, chest is emptied quickly.

Sensation as of a great stone laid upon the heart.

Sensation (soon after) as if the thoracic wall anterior to heart were broken out or torn away.

Pulse sharp.

Desire to remove clothing from chest.

Pain in chest and both arms.

*Neck, Back, etc.*—Painful sensation in the sides of the neck, left, at mastoid or below it, continuing longer than on right side.

Pain in left neck behind mastoid process, running backward and upward.

Pain through right shoulder blade (scapula).

Dorsal vertebræ feel painful (third day).

Tenderness along spines of cervical and upper dorsal vertebræ (fourth day).

Pain in muscles of thorax midway between scapula and sacrum (sixth day).

Pain on pressure of muscles of left side of the neck.

Back lame on stooping.

Pain in right scapula.

Pain in neck.

Pain in left side above and along clavicle.

Fatigue in lumbar region on riding.

*Upper Extremities.*—Tired feeling in both arms (second day).

Drawing pain in index finger of both hands.

Pain in both upper arms.

Pain running across inner side of left arm, felt longest at bend of the elbow.

Pain in left shoulder like that produced by carrying a heavy load.

Pain running along the back down to the arms.

Dull pain in left elbow and forearm.

Pain with numbness in left forearm, ulnar side (third day).

Pain along inner side of right upper arm.

Pain with numbness of right arm while writing.

Pain in metacarpal bone of right thumb.

Pain (very noticeable) in metacarpal phalangeal joint of right hand.

Lameness in right forearm above wrist.

Drawing from end of right thumb upward, pain quite constant.

Considerable soreness on contact of anterior muscles of right arm.

Pain on ulnar side of left carpo-metacarpal joint (fourth day).

Pain in external border of left elbow joint.

Pain at and back of left shoulder joint.

Lameness of left little finger.

Pain over ulna posteriorily.

Pain above wrist.

Tenderness of the flexor muscles of both upper arms.

Pain in right ring finger at 3 P. M. and repeated (fifth day).

Pain at junction of second and third phalanx (last joint) of left index finger.

Pain in dorsum of right hand.

Pain in left forearm.

Pain in both arms and chest.

Pain about the corocoid process of right shoulder.

Pain in third phalanx of left index finger.

Pain in right little finger running through bone.

Pain in right ring finger.

Pain in right wrist.

Pain in first and second metacarpal bones (sixth day) of right hand.

Pain in the dorsum of left hand.

Pain in left little finger.

Pain on back of left wrist, running to forearm.

Pain in the anterior muscles of upper arm.

*Lower Extremities.*—Pain in right knee (second day).

Pain through right hip (fifth day).

Pain in right great trochanter.

Pain on the inner side of left knee (repeated).

Pain on left knee, inner and lower border.

Pain in both knees.

Pain in both knees on rising.

Pain in hamstring tendons of left thigh.

Pain in right hip (sixth day).

Pain in head of the right thigh bone.

Pain in right patella, very sore, difficult to touch without very considerable pain.

Pain above right external malleolus.

Pressing or pressive feeling beginning at the sacrum and running down through both thighs down to feet.

Pain in different joints of the lower extremities.

*Skin.*—Itching of the nose (second day).

Itching on various parts of the body (general itching) (third day).

Itching pustule of face near right ala of nose.

Itching of the right popliteal space, with roughness of the skin (fifth day).

Profuse shedding of the hair on combing the head.

Itching with roughness of the skin of a spot a few inches square above left knee.

Itching of a spot a few inches below left scapula, with a condition of the skin like eczema periodically.

*Sleep.*—Not sleeping late at night.

Not sleeping at 11 P. M., mind disturbed (fourth day).

Dreamed of dogs (fifth day).

Dream of a fracas which caused great excitement in the dreamer.

Drowsiness at 11 P. M. (sixth day).

Drowsiness (third day).

Slept pretty well (fifth day).

Awakes at 5 A. M. (sixth day).

Awakes at 9 A. M. (seventh day, Sunday).

Recurrence of old dreams of years ago.

Yawning (second day).

*Generalities.*—Feeling miserably on retiring.

Throws himself on bed without undressing.

Great yawning fit (third day).

Feels not pleasant.

Feels half sick.

Very dull in the morning, all morning.

Feels very badly, has an ill-defined bad feeling in the evening and at night.

Easily chilled in a room; better on disrobing for bed.

Alternation of symptoms of mind and bodily pains. When pains of the body are noticed, symptoms affecting the mind are suspended. The mind loses its characteristics, is clear, and one feels better.

### Remarks.

In looking over the above proving we find a number of illustrations of the alternate action of the drug. But perhaps what strikes the reader most forcibly is the way the symptoms follow Reuter's series. The most prominent symptoms early developed, catarrhal and gastric, have come and gone within three or four days, while those affecting the chest, heart, sensorium, eyes, brain, and nerves are more slowly developed, and are the ones that persist. Another thing to be noticed is the long duration of its action. The high-water mark in regard to its action was not reached (I mean its action on the nervous system) until nearly ten days after discontinuing to take it. It is an *anti-psoric* of remarkable power. Some skin symptoms developed by it persisted off and on for years, two or three of which I will mention. "Itching of the right popliteal space," this after continuing for eight or nine years disappeared. I think some Sepia$^{1m}$ I took had something to do with its disappearance. Another: "Itching with roughness of the skin, like eczema, above the left knee anteriorly." This still persists. I still have "Itching, with an eruption resembling at times herpes zoster below the left scapula." This is still present, although annoying, I have done nothing to cause its disappearance.

In regard to *verifications* I could report a goodly number. One of the first I ever had was a case of eczema of both hands, extending as far as the elbows. Cured in six weeks. The provings point in the direction of kidney troubles, and I have seen it speedily cause the disappearance of deposits in the urine that were giving much inconvenience. In a case of dropsy of cardiac and renal origin (albuminuria) in which there was great

œdema, cured in two or three weeks. Sleeplessness, peculiar in its nature, corresponding to the proving, is relieved by it. Intercostal neuralgia, especially on left side. Anterior crural neuralgia, an aggravated case, promptly relieved. I need not say that the symptoms strongly point to rheumatism. I could say much on that part of the subject, and there is the sphere in which it has seemed to have been useful by the professional friend to whom I have furnished the medicine for trial. In a monograph by Dr. R. E. Kunge, of New York, and the writer, I ventured the prediction that Cereus Bonplandii would prove of value in the treatment of insanity. I send you the report of two cases. I have one other still under treatment. A patient for fourteen years in the Middletown Insane Hospital, improving, called to see Ida Reamer, a young woman of eighteen, living in New Scotland, on what is called the Heldeberg Mountain or hill, on the evening of April 19th, 1884. For some time previously she had been living with a relative in Albany, attending school and assisting in household labor. Had studied hard and probably over-exerted her strength. Her friends noticing that she was not her former self, and that though usually amiable and cheerful, she had become gloomy and taciturn, brought her home. Rest did her no good, and I was called after she had been home for some time. On my visit I noticed she would not answer questions; was wandering aimlessly about the house; could not sit still, if seated, more than a few minutes. During my visit I think she changed her position a dozen or fifteen times. She would go to the water pail and get a drink, then in a minute or two would get up and go to the door. After standing a minute or two she would come in and sit down, only to rise up and repeat her restless wanderings. I could elicit nothing from the mother of anything wrong in regard to the menstrual function. Prescribed Cereus Bonplandii, fourth decimal. Did not call again, but was informed by her friend that she soon regained her health. Was requested to call again to see Ida R. on November 29th of the same year. This time there was considerable mental disturbance; she had attended some entertainment which she had considered of a questionable nature, and had been worrying over it. Although living out at service, it did not appear that she had overworked. I found her sitting still; she would sit for hours. If any one disturbed her she would curse, swear, throw boots and shoes or anything that came in her way, resisted attempts made by her friends to remove her to her home. Prescribed Cer-bon.[4]. Saw her December 3d, 7th, 10th, at the end of which time she was entirely free from any mental manifestations, and although under observation, has never experienced a return of them to the present date.

In the summer of 1879 was consulted in the case of Mrs. D. V. afflicted with melancholia for a year or two. The disease had appeared just subsequent to her confinement with her last child. Prescribed wholesome advice in regard to mode of life, etc., and very little medicine. In a few months she was apparently as well as ever. June 5th, 1884, was called to see Mrs. D. V. She had quite recently given birth to a child, and was developing delusions, most of which were those of a spiritual nature. She thought she had committed the unpardonable sin, or that she had offended some of her friends, and was constantly worrying. Appetite very poor. Prescribed Cer-bon.[4], gave her nourishing diet with Maltine and Pepsin to assist digestion. On July 11th she was about the house attending to her household duties.

Adjourned to 8 P. M.

---

*Second Day—Evening Session.*

Wednesday, June 22d, 8 P. M.

